# Canine peripheral blood TCRαβ T cell atlas: Identification of diverse subsets including CD8A^+^ MAIT-like cells by combined single-cell transcriptome and V(D)J repertoire analysis

**DOI:** 10.3389/fimmu.2023.1123366

**Published:** 2023-02-23

**Authors:** Maria Eschke, Peter F. Moore, Haiyang Chang, Gottfried Alber, Stefan M. Keller

**Affiliations:** ^1^ Institute of Immunology/Molecular Pathogenesis, Center for Biotechnology and Biomedicine, College of Veterinary Medicine, University of Leipzig, Leipzig, Germany; ^2^ Department of Pathology, Microbiology and Immunology, School of Veterinary Medicine, University of California, Davis, CA, United States; ^3^ Department of Mathematics and Statistics, University of Guelph, Guelph, ON, Canada

**Keywords:** dog, canine T cells, TCRαβ, single-cell RNA-sequencing, immune repertoire, peripheral blood, MAIT-like cells, innate-like T cells

## Abstract

The dog is valued as a companion animal and increasingly recognized as a model for human disorders. Given the importance of T cells in health and disease, comprehensive knowledge of canine T cells can contribute to our understanding of pathogenesis mechanisms and inform the development of new treatment strategies. However, the diversity of canine T cells is still poorly understood mainly due to the lack of species-reactive antibodies for use in flow cytometry. The aim of this study was to generate a detailed atlas of peripheral blood TCRαβ^+^ T cells of healthy dogs using single-cell RNA-sequencing (scRNAseq) combined with immune repertoire sequencing. A total of 22 TCRαβ^+^ T cell clusters were identified, which were classified into three major groups: CD4-dominant (11 clusters), CD8A-dominant (8 clusters), and CD4/CD8A-mixed (3 clusters). Based on differential gene expression, distinct differentiation states (naïve, effector, memory, exhausted) and lineages (e.g. CD4 T helper and regulatory T cells) could be distinguished. Importantly, several T cell populations were identified, which have not been described in dogs before. Of particular note, our data provide first evidence for the existence of canine mucosa-associated invariant T cell (MAIT)-like cells, representing one of three newly identified FCER1G^+^ innate-like CD8A^+^ T cell populations in the peripheral blood of healthy dogs. In conclusion, using scRNAseq combined with immune repertoire sequencing we were able to resolve canine TCRαβ^+^ T cell populations at unprecedented resolution. The peripheral blood TCRαβ^+^ T cell atlas of healthy dogs generated here represents an important reference data set for future studies and is of relevance for identifying new targets for T cell-specific therapies.

## 1 Introduction

An in-depth understanding of the physiological and pathophysiological processes in different species is of increasing importance in the fields of vaccinology and immunotherapy. Considering interspecies differences of immune cells, it is essential to unveil species-specific characteristics. Several features of dogs (*Canis lupus familiaris*) make this species an attractive candidate for immunotherapy research. As companion animals, dogs are largely exposed to the same environmental conditions as humans and develop common human health disorders, including autoimmune diseases (1), allergies (2), and cancer (3). T lymphocytes expressing the T cell receptor (TCR) αβ are well-known to play a central role in adaptive immunity across species and are therefore promising targets for immunotherapies. However, the diversity of canine TCRαβ^+^ T cells is still incompletely understood, primarily due to the paucity of dog-reactive immunophenotyping reagents for use in flow cytometry as well as insufficient *a priori* knowledge of marker genes. Single-cell RNA-sequencing (scRNA-seq) enables unbiased, high throughput and high-resolution transcriptomic analysis of heterogeneous cell populations with a multitude of applications across biomedical sciences (4). In non-traditional model organisms, scRNA-seq has been shown to present a powerful tool to characterize the cellular diversity of immune cells (5–7).

In the canine species, scRNA-seq has been used previously to identify cellular populations of the bronchoalveolar lavage fluid of healthy dogs (6) and to evaluate the expression patterns of SARS-CoV-2 entry factors in lung cells (8). However, both studies neither resolved CD4^+^ and CD8^+^ T cell subpopulations nor analysed the immune repertoire.

Here, we used the potential of scRNA-seq in combination with immune repertoire sequencing to generate a highly detailed atlas of peripheral blood TCRαβ^+^ T cells of healthy dogs. A total of 22 TCRαβ^+^ T cell clusters were identified and divided into three major groups: (i) a CD4-dominant group containing 11 clusters, (ii) a CD8A-dominant group containing eight clusters, and (iii) a CD4/CD8A-mixed group containing three clusters. Besides well-known CD4 and CD8A T cell differentiation states (naïve, effector, memory) and lineages (e.g. T helper and regulatory CD4 T cells), our comprehensive analysis revealed several novel T cell subtypes not previously identified in dogs, including a MAIT-like population characterized by a highly restricted TCR repertoire. The combined transcriptome and V(D)J immune-repertoire analysis provided important reference data highlighting the cellular heterogeneity of peripheral blood TCRαβ^+^ T cells of healthy dogs. It can be referenced in future experiments to elucidate tissue-specific properties of the individual T cell populations under both physiological and pathophysiological conditions.

## 2 Materials and methods

### 2.1 Dogs, blood sample collection

From 4 healthy experimental Beagle dogs (**Table 1**, one female, three male, age: 5 – 9 years) of the Faculty of Veterinary Medicine (Leipzig University, Leipzig, Germany), venous blood was taken by venipuncture of the *vena cephalica antebrachii* into heparinized vacutainer tubes (BD Vacutainer^®^, 10 ml, Li-Heparin 17 IU/ml Becton Dickinson, Heidelberg, Germany). All dogs received routine vaccinations against canine distemper, rabies, canine infectious hepatitis, parvovirus infection, parainfluenza, and leptospirosis. The study was authorized by the Saxony State Office (*Landesdirektion Sachsen*) in Leipzig, Germany (approval number: DD24.1-5131/444/30).

**Table 1:** Subject characteristics as well as sequencing and mapping quality control metrics for each dog.

Study subject	Sex	Age (years)	Estimated number of cells	Reads mapped confidently to:		Median genes per cell	Median UMI counts per cell	Total genes detected
				Genome	Transcriptome			
DOG 1	F	5	7,428	72.3%	41.0%	1,394	3,534	13,649
DOG 2	M	7	8,873	73.2%	42.3%	1,534	3,844	13,849
DOG 3	M	7	8,402	72.5%	41.2%	1,498	3,716	13,720
DOG 4	M	9	8,422	73.3%	42.1%	1,561	3,809	13,667

### 2.2 Isolation of canine peripheral blood mononuclear cells

PBMC were isolated by density gradient centrifugation. Briefly, blood was diluted at a ratio of 1:1 with phosphate buffered saline (PBS), layered above Biocoll Separating Solution (density 1077 g/l, Biochrom AG, Berlin, Germany) and centrifuged at 500 x g for 30 min at room temperature (RT) without brake. PBMC at the interphase were harvested into PBS and centrifuged at 500 x g for 10 min at RT. After another washing step with PBS, erythrocytes were lysed by incubation in 150 mM NH_4_Cl, 8 mM KHCO_3_, 2 mM EDTA (pH 7) for 5 min at RT. The reaction was stopped by addition of PBS containing 3% fetal bovine serum (FBS, Thermo Fisher Scientific, Carlsbad, USA; and PAN-Biotech, Aidenbach, Germany). After washing with PBS, PBMC were counted in Trypan blue (Sigma-Aldrich, Taufkirchen, Germany) using a hemocytometer (Laboroptik, Lancing, UK).

### 2.3 Fluorescence-activated cell sorting of TCRαβ^+^ T cells

**Figure 1:**
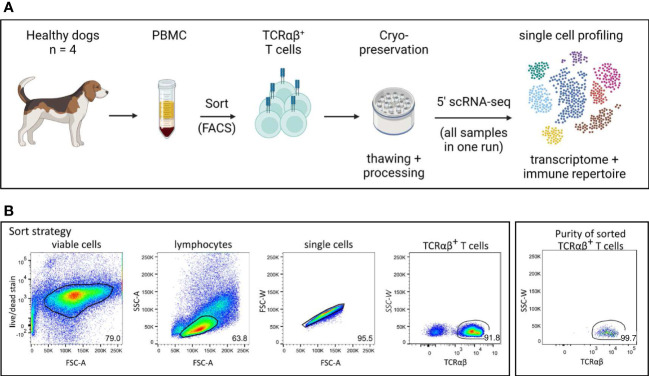
Experimental setup for single cell analysis of canine TCRαβ^+^ T cells. **(A)** Peripheral blood mononuclear cells (PBMC) of four healthy Beagle dogs were used. Cryopreserved TCRαβ^+^ T cells isolated by fluorescence-activated cell sorting (FACS) were applied to 5’ single-cell RNA sequencing (5’ scRNA-seq) for integrated transcriptome and TCR repertoire analysis. (created with BioRender) **(B)** The gating strategy for isolation of TCRαβ^+^ T cells by FACS as well as the purity of the sorted population are shown (representative data of one dog). Numbers in plots imply percentages.

PBMC were first stained with the fixable viability dye eFluor 780 (Thermo Fisher Scientific, Carlsbad, USA) according to the manufacturer’s protocol to discriminate dead from viable cells. In a second step, they were incubated with a mixture of heat-inactivated normal serum derived from dog and rat (each 15% in PBS) to block unspecific binding of Fc receptors. Then, cells were incubated with anti-canine TCRαβ (clone CA15.8G7) hybridoma supernatant, for 15 min in the dark on ice. A PerCP/Cy5.5-conjugated goat-anti-mouse IgG secondary antibody (Biolegend, San Diego, USA) was used for detection. Sorting was performed using the BD FACSAria™ III flow cytometer (Becton Dickinson, Heidelberg, Germany). The sorting strategy is shown in **Figure 1B**. After exclusion of dead cells and doublets, TCRαβ^+^ T cells from the lymphocyte gate were sorted with a purity >99% (Re-analysis using the FlowJo™10 software (Treestar Inc., Ashland, OR, USA). Isolated TCRαβ^+^ T cells were cryopreserved in 90% FBS, 10% dimethyl sulfoxide (Sigma-Aldrich) and shipped on dry ice to the Institute of Pathology, Microbiology & Immunology of the UC Davis.

### 2.4 Single-cell 5’ RNA and V(D)J sequencing

All 4 samples were thawed at once so that scRNA-Seq could be performed in a single run to avoid batch-specific effects. After confirmation of cellularity (1.1-1.4 x 10^6^ cells/ml) and viability (78-90%) of TCRαβ^+^ T cells, transcriptome and V(D)J libraries were prepared at the UC Davis Genome Center. Briefly, ~10,000 TCRαβ^+^ T cells per lane were loaded on a Chromium Single Cell Controller (10× Genomics, Pleasanton, CA, USA). Within individual gel beads in emulsion (GEMs), captured cells were lysed and the released RNAs were barcoded through reverse transcription. Starting mRNA molecules were indexed using Unique Molecular Identifier (UMI). Following emulsion breakage, barcoded cDNA was amplified and libraries were constructed using the Chromium Next GEM Single Cell 5’ Kit v2 (10x Genomics). The canine T cell receptor alpha (TRA) and T cell receptor beta (TRB) genes were amplified using the Chromium Single Cell V(D)J Enrichment Kit according to the manufacturer’s instructions but with primers adapted for the canine genes (see **Supplementary Table 1** for primer sequences and additional information). Libraries were sequenced using the Illumina NovaSeq S4 platform using 150 paired-end reads to a target read depth of 30,000 reads per cell. Single-cell data were pre-processed using the Cell Ranger software 5.0.1 (10x Genomics). The standard workflow was used for alignment of reads to the reference genome (CanFam 3.1 annotation genebuild updated 2019-06, GenBank assembly accession: GCA_000002285). Reads were organized by cell barcodes and UMI counting resulting in a digital expression (gene-cell barcode) matrix. scRNA-seq data are available in the NCBI GEO repository, accession number GSE218355.

### 2.5 Transcriptome data analysis

The digital gene expression matrix resulting from data preprocessing was analyzed using the R package Seurat v4.1.1 (9, 10). Analysis R code is available upon request. Briefly, genes expressed in fewer than three cells per subject were excluded. Data from all four dogs were merged, and cells with less than 500 or more than 20,000 UMIs (transcripts) as well as low quality/dying cells with greater than 10% UMIs assigned to mitochondrial genes were excluded. Following filtering, the global-scaling normalization method “LogNormalize” was applied, and variable genes were set to all genes after filtering.

Principal component analysis (PCA) was used for dimensionality reduction and unsupervised graph-based clustering (resolution factor 1.61) was performed on the first 50 principal components. Different resolution factors were tested and cluster stability was evaluated using the clustree package (11). Additional subdivisions of clusters at higher resolutions were not associated with further detection of informative differentially expressed genes. The data were visualized by non-linear dimensional reduction using uniform manifold approximation and projection (UMAP) plots. The FindAllMarkers function was used to identify differentially expressed genes (DEGs) across clusters (**Supplementary Table 2**). Some DEGs not annotated in the reference genome were further blasted on the Ensembl genome browser v108) (12) for dog species to increase the annotation rate. Cell clusters were manually annotated based on expression of T cell signature genes, primarily known from human and mouse studies. ViolinPlots, DotPlots, and FeaturePlots were used for visualizing differences in gene expression between clusters.

### 2.6 V(D)J data analysis

All basic analyses were done using the R software (version 4.1.2) and the following packages: circlize (chord diagrams), ggseqlogo (sequence logos), igraph (centrality), RColorBrewer (colors), stringdist (Hamming distance), tidy code (tidyverse). To identify clusters in the TRA and TRB datasets, junctional amino acid sequences were first stratified by V and J gene subgroups followed by hierarchical clustering using the single and complete linkage methods and a cut-off of one Hamming Distance (HD). This resulted in clusters comprising one or multiple clonotypes. All clusters were numbered, regardless of how many clonotypes they contained. Pairwise amino acid comparison and hierarchical clustering were done using the Python software (version 3.10.2).

### 2.7 Statistical analysis

Differential gene expression was measured using non-parametric Wilcoxon rank sum tests adjusted for multiple testing with Bonferroni correction. Only significant DEGs with an adjusted *P*-value < 0.05 were retained.

## 3 Results

### 3.1 Single-cell RNA-sequencing of canine TCRαβ^+^ T cells resolves CD8A-dominant, CD4-dominant, and CD4/CD8A-mixed clusters

**Figure 2:**
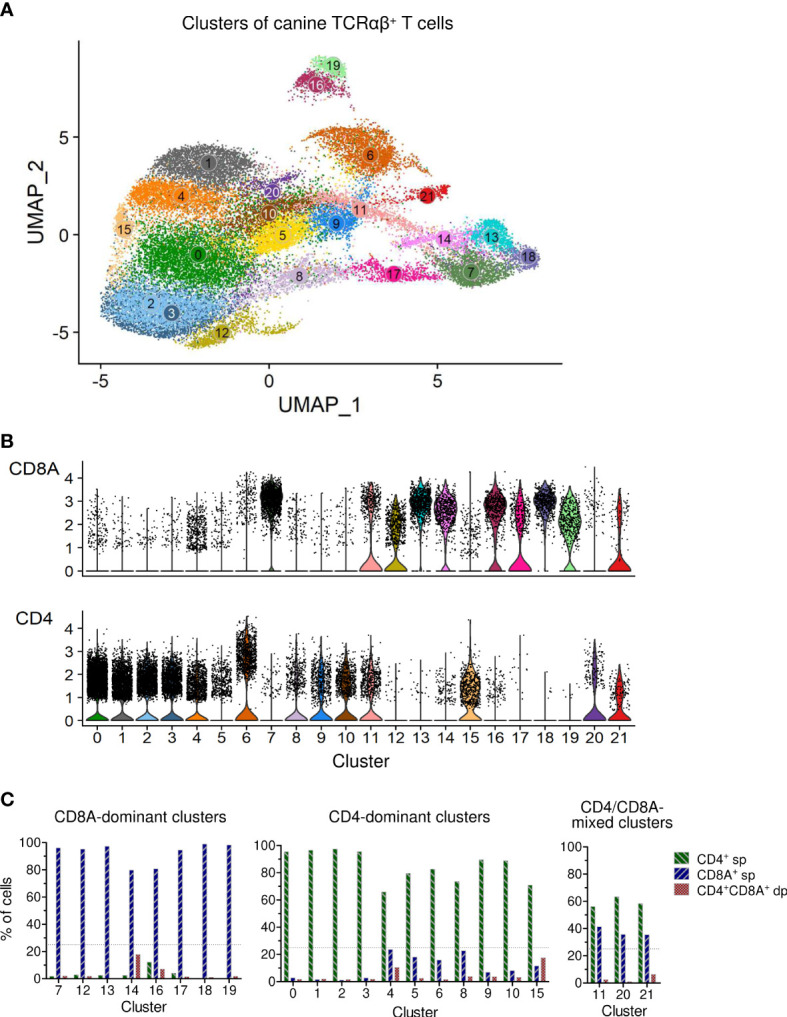
Single-cell RNA-sequencing analysis of TCRαβ^+^ T cells purified from peripheral blood of healthy dogs revealed various CD8A-dominant, CD4-dominant and CD4/CD8A-mixed clusters. **(A)** Unsupervised graph-based clustering of merged data from all four dogs yielded 22 Clusters of canine TCRαβ^+^ T cells. **(B)** Visualization of CD8A and CD4 expression by violin plots. **(C)** The percentage of CD8A^+^ single-positive (sp), CD4^+^ sp and CD4^+^CD8A^+^ double-positive (dp) T cells of each cluster was determined by virtual gating after exclusion of CD4^-^CD8A^-^ double-negative cells. Based on the CD8A/CD4 expression pattern, clusters were devided into three major groups: (1) CD8A-dominant (n = 8: C7, C12-14, C16-19), (2) CD4-dominant (n=11: C0-C6, C8-C10, C15), and (3) CD4/CD8A-mixed clusters (n = 3: C11, C20-21), mainly composed of CD8A^+^ and CD4^+^ sp cells. Dotted line: treshold for maximum CD4^+^/CD8A^+^ sp cells in CD8A/CD4-dominant clusters.

In this study, we used combined transcriptome and V(D)J analysis at the single cell level to establish a detailed baseline TCRαβ^+^ T cell atlas of the dog. Cryopreserved viable TCRαβ^+^ T cells isolated with high purity (>99%) from peripheral blood of four healthy Beagle dogs by fluorescence-activated cell sorting (FACS) were applied to 5’ single-cell RNA sequencing analysis (5’ scRNA-seq) (**Figure 1**). Sequencing and mapping quality control metrics for each dog are summarized in **Table 1**. Unsupervised clustering of 31,909 cells from four dogs was performed for different resolution levels and a final resolution with 22 clusters was chosen. (**Figure 2A**). All clusters were present in each individual dog (**Supplementary Figure 1**). Violin plots visualizing CD8A and CD4 expression indicated the presence of CD8A^+^, CD4^+^ and CD8A^+^/CD4^+^ mixed clusters (**Figure 2B**). However, especially clusters in which CD4 was detected also contained a considerable proportion of cells without CD4 expression (**Figure 2B**), a phenomenon already observed in previous scRNA-seq studies using equine or human PBMC (5, 13). The number of TCRαβ^+^ CD8α^-^CD4^-^ double-negative (dn) T cells detected by scRNA-seq was higher than what would be expected based on previous flow cytometric analyses [~15% (14)]. To better characterize the proportion of CD8A/CD4 expression in each cluster, CD8A^-^CD4^-^ dn cells were excluded and the percentage of CD8A^+^ single-positive (sp) cells, CD4^+^ sp cells, and CD4^+^CD8A^+^ double-positive (dp) cells of each cluster was evaluated (**Figure 2C**). Eight clusters consisting mainly of CD8A sp T cells were detected (C7, C12-C14, C16-C19). The proportion of CD8A^+^ sp cells within those clusters was 95-99% except for clusters 14 and 16 (~80% CD8A^+^ sp), which also contained CD4 sp (C16: ~12%) and/or CD4^+^CD8A^+^ dp T cells (C14: 18%, C16: 7%). Therefore, clusters C7, C12-C14, and C16-C19 were classified as CD8A-dominant. Besides clear-cut CD4 clusters with 89-98% of the cells showing a CD4 sp phenotype (C0-C3, C9, C10), several clusters showed dominance of CD4^+^ sp cells but additionally comprised >10% CD8A^+^ sp and/or CD4^+^CD8A^+^ dp T cells. Of those, clusters consisting of ≥66% CD4 sp T cells and <25% CD8A^+^ sp cells were classified as CD4-dominant (C4-C6, C8, C15). The remaining three clusters (C11, C20-C21) with 35-41% CD8A^+^ sp cells and 56-63% CD4^+^ sp cells were classified as CD4/CD8A-mixed. Notably, only a small proportion (1-6%) of cells within the latter group had a CD4^+^CD8A^+^ dp phenotype (**Figure 2C**).

Taken together, based on the proportions of CD8A^+^ and CD4^+^ cells, clusters were divided into three major groups: (i) a CD4-dominant group containing eleven clusters (C0-C6, C8-C10, C15), (ii) a CD8A-dominant group containing eight clusters (C7, C12-C14, C16-C19), and (iii) a CD4/CD8A-mixed group containing three clusters (C11, C20, C21) (**Figure 2C**).

### 3.2 CD4-dominant clusters contain known T helper/T regulatory subsets as well as subsets previously unrecognized in dogs

**Figure 3:**
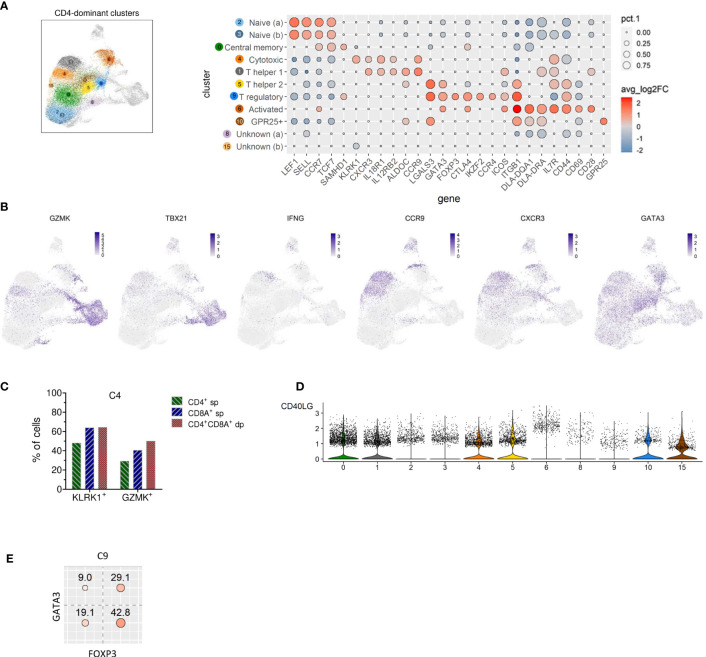
Identification of naive, memory and known T helper and regulatory subsets among canine TCRαβ^+^ CD4-dominant clusters and detection of CD4 T cells with cytotoxic potential. **(A)** CD4 cell-dominat clusters highlighted in UMAP plot were annotated based on expression of T cell subset marker genes shown in Dot plot. The dot size corresponds to the percentage (pct.1) of cells expressing the gene in each CD4-dominant cluster, the color represents the expression level (average log2 fold change). Genes were ordered to visualize the differences/similarities between cell types. **(B)** Expression pattern of select genes across all canine TCRαβ^+^ clusters visualized by FeaturePlots **(C)** Within C4, expression of the cytotoxic effector molecules KLRK1 and GZMK was detected in the dominating CD4^+^ sp cells and in the minor fractions of CD8A^+^ sp and CD4^+^CD8A^+^ dp T cells by virtual gating after exclusion of CD4^-^CD8A^-^ double-negative cells. **(D)** Visualization of CD40LG expression in CD4-dominant clusters by violin plots. **(E)** Virtual gating for FOXP3 and GATA3 within C9 (T regulatory CD4-dominant cluster) reveals co-expression of the transcription factors in ~30% of the cells.

Clusters of the three major groups of TCRαβ T cells were manually annotated by assessing the expression of lineage-defining marker genes. Two naïve TCRαβ^+^ CD4 T cell clusters (C2, C3) were identified based on elevated expression of the lymph node homing receptors CCR7 and SELL (encoding CD62L/L-Selectin) as well as of the transcription factors TCF7 and LEF1 (13) (**Figure 3A**). As expected, the frequency of naïve CD4 T cells was lowest in the oldest and highest in the youngest dog (**Table 2** and **Supplementary Figure 1**).

**Table 2:** Total number of cells of each TCRαβ^+^ T cell cluster and their distribution among dogs.

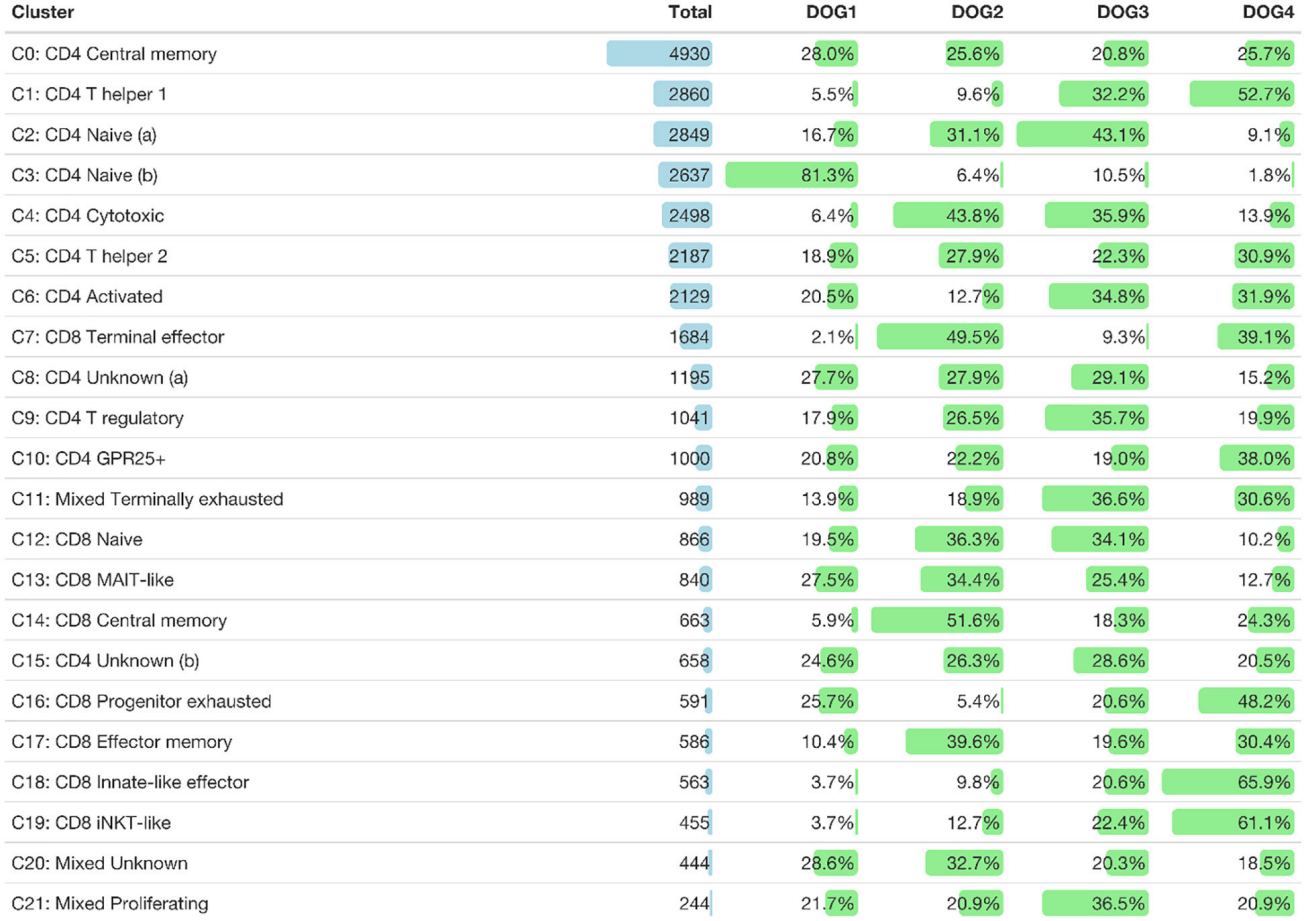

A direct comparison between the two clusters containing naïve CD4^+^ T cells revealed that C2 expressed higher levels of quiescence-associated genes HOMER2, CCR7, SELL, KLF3, and IL7R (15–18) compared to C3 (**Supplementary Figure 2** and **Supplementary Table 3**). Among the genes with higher expression in C3 compared to C2 were genes encoding interferon-induced transmembrane proteins (IFITM) and metallothionin (MT) (**Supplementary Figure 2** and **Supplementary Table 3**). IFITM proteins as well as MT have been shown to be involved in differentiation of naive CD4 T cells (19, 20). Whereas C2 is more abundant than C3 in dogs 2, 3, and 4, the opposite was true for the youngest dog (dog 1) (**Table 2** and **Supplementary Figure 1**).

C0, which was similarly abundant in all dogs (**Table 2** and **Supplementary Figure 1**), was identified as resting central memory CD4 T cell cluster based on elevated expression of CCR7, TCF7 and SAMHD1 (13, 21–23) (**Figure 3A**). C4 was characterized by increased expression of the killer cell lectin-like receptor K1 (KLRK1) encoding NKG2D, a prototypical activation marker on human and mouse NK cells mediating cytotoxicity (24, 25). Cytotoxic potential of cells within C4 was further supported by expression of the cytotoxic effector molecule granzyme K (GZMK), which was evident when C4 was compared against all other CD4-dominant clusters (**Figure 3B** and **Supplementary Table 4**). However, albeit classified as a CD4-dominant cluster, C4 also comprised CD8A^+^ sp cells and CD4^+^CD8A^+^ dp cells (**Figure 2C**). To verify that CD4^+^ sp cells in C4 contributed to the cytotoxic signature in this cluster, after exclusion of CD4^-^CD8^-^ dn cells, CD4^+^ sp, CD8A^+^ sp and CD4^+^CD8A^+^ dp cells were analyzed for expression of GZMK and KLRK1 separately. Indeed, cytotoxicity-associated genes were detected in all three fractions (**Figure 3C**) supporting the notion that cytotoxic CD4^+^ T cells exist in dogs. Cytotoxic CD4^+^ T cells are well described in mice and humans in the context of viral infections, autoimmunity, and cancer (26, 27). In humans, NKG2D^+^ highly activated cytotoxic CD4^+^ T cells have been associated with immune senescence (26, 28, 29). Although the low number of dogs used does not allow definite conclusions, an association of canine cytotoxic CD4^+^ T cells with age was not supported by this study, as dog 1 (youngest) and dog 4 (oldest) had a lower frequency of cytotoxic CD4^+^ T cells than dogs 2 and 3 (**Table 2** and **Supplementary Figure 1**).

Distinct populations of CD4^+^ T helper (Th) cells were resolved by scRNA-seq in this study. In C1, elevated expression of genes associated with the Th1 phenotype was detected, including IL18R1, CXCR3, IL12RB, and ALDOC (30–34). Although the master transcription factor of Th1 cells T-bet (encoded by TBX21) did not appear as DEG in C1, there was a trend of weak TBX21 expression among CD4-dominant clusters in C1 and C4 matching the Th1/cytotoxic CD4 phenotype (**Figure 3B**). Of note, low levels of TBX21 transcripts in canine CD4^+^ T cells is consistent with our flow cytometric data suggesting IFN-γ production despite weak T-bet expression (35). Indeed, constitutive expression of IFNG encoding the Th1-effector cytokine was detected in C1. Noteworthy, CCR9, which, in addition to its role as gut homing molecule, has been shown to shape immune responses by inhibiting development of regulatory T (Treg) cells (36) appeared as top DEG of the CD4-dominant clusters C1 (Th1) and C4 (cytotoxic CD4^+^ T cells) (**Figure 3B**). Individual differences were observed with respect to the proportions of these two clusters: In dog 1 (youngest), they only comprised few cells. Dog 4 (oldest) contained a large Th1 (C1) and rather small cytotoxic CD4^+^ (C4) cluster, whereas the opposite was found for dog 2. In dog 3 both clusters represented large populations (**Table 2** and **Supplementary Figure 1**).

C9 was identified as distinct Treg cluster expressing the marker genes FOXP3, CTLA4, IKZF2, and CCR4 (**Figure 3A**). Interestingly, FoxP3 and GATA3 were co-expressed in ~30% of cells in C9 (**Figure 3E**), confirming our previous flow cytometry results showing co-expression of both transcription factors in small fractions of canine CD4^+^ T cells and CD4^-^CD8A^-^ dn T cells (14). In mice, co-expression of GATA-3 has been shown to stabilize FoxP3 expression and to prevent conversion of CD4^+^ FoxP3^+^ T cells into a pro-inflammatory Th17 cell phenotype (37–39).

Based on elevated GATA3 expression but lack of FoxP3, C5 was annotated as Th2 cell cluster (**Figure 3A**). Both, clusters 5 and 9 are characterized by expression of Galectin-3 (encoded by LGALS3). A Th2-promoting activity of Galectin-3 as well as its expression on Treg cells have been shown previously (40).

Interestingly, increased expression of LGALS3 was also found in a third cluster (C10). C10 is characterized by expression of GPR25, which encodes an orphan G protein-coupled receptor. CD4^+^ GPR25^+^ cells represent another previously unrecognized canine TCRαβ^+^ T cell population.

Cluster 6 comprises activated TCRαβ^+^ CD4^+^ T cells defined by high expression of the early activation marker CD69 as well as of CD44, CD28, and MHC-II molecules (DLA-DRA, DLA-DQA1) (**Figure 3A**). Notably, C6 is heterogenous in terms of e.g. GATA3 and CXCR3 expression (**Figure 3B**), indicating, that it contains recently activated T cells with different effector functions.

Two CD4-dominant clusters (C8, C15) were designated “unknown” CD4^+^ T cells due to the lack of lineage-defining signature transcripts (**Figure 3A**). Increased expression of transcripts from mitochondrial genes in C8 may indicate clustering of cells with reduced viability, which could have resulted from freezing/thawing.

Interestingly, elevated expression of CD40LG, a cross-species marker of T helper cell (Th) activation by antigens (41–44), was detected in C5 (Th2) (**Supplementary Table 2** and **Figure 3D**). Furthermore, a trend towards a higher expression level of CD40LG is observed in C6, supporting recent activation (**Figure 3D**).

### 3.3 Canine TCRαβ^+^ CD8A-dominant clusters represent distinct differentiation states and include three innate-like subsets characterized by high expression of FCER1G

**Figure 4:**
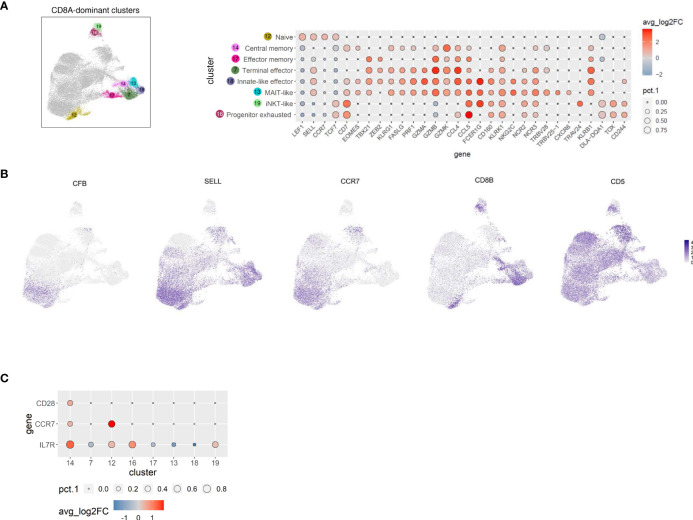
TCRαβ^+^ CD8A-dominant clusters of the dog comprise distinct differentiation states and three innate-like subsets characterized by high expression of FCER1G, including a MAIT-like cell type. **(A)** CD8A-dominant clusters highlighted in UMAP plot were annotated based on expression of T cell subset marker genes shown in Dot plot. The dot size corresponds to the percentage (pct.1) of cells expressing the gene in each CD8A-dominant cluster, the color represents the expression level (average_log2 fold change). Genes were ordered to visualize the differences/similarities between cell types. **(B)** Expression of select genes across all canine TCRαβ^+^ clusters visualized by FeaturePlots **(C)** Direct comparison of C14 with all other CD8A-dominant clusters confirms the assignment to central memory T cells.

C12 comprises naïve CD8A^+^ T cells that share a similar gene expression profile with the two naïve CD4 clusters C2 and C3 (LEF1, SELL, CCR7, TCF7) (**Figures 3A**, **4A**), which are also located closely in the UMAP plot (**Figure 2A**). Furthermore, complement factor B (CFB) was found to be associated with canine TCRαβ^+^ naïve CD4 and CD8A T cells (**Figure 4B**). Whereas naïve human CD8^+^ T cells express KLRK1 encoding the activating NK receptor NKG2D (45), our data suggest activation-induced transcription in canine CD8^+^ T cells (**Figure 4A**), that is also observed in mice (45).

Cells in C14, which were characterized by expression of CD7 and EOMES (**Figure 4A**), additionally showed elevated expression of the memory markers IL7R, CCR7, and CD28 when compared with all other CD8A-dominant clusters (**Figure 4C** and **Supplementary Table 5**), reminiscent of a central memory phenotype (46). Noteworthy, expression of SELL was not restricted to naïve and central memory CD8A clusters, but was also detected in CD8A clusters containing effector cells with high expression of cytotoxic molecules (C7, C13, C18) (**Figures 4A, B**). In contrast, CCR7 expression was found to be specifically associated with the naïve/central memory phenotype in CD8A-dominant clusters (C12, C14) (**Figures 4B, C**).

C17 expressed multiple effector transcripts (ZEB2^+^, GZMB^+^, GZMK^+^, CCL4^+^, and CCL5^+^), consistent with effector memory CD8 T cells, while C7 showed a signature of terminally differentiated effector CD8 T cells (ZEB2^+^, KLRG1^+^, PRF1^+^, FASLG^+,^GZMA^+^, GZMB^hi^, GZMK^+^, CCL4^hi^, CCL5^+^) (**Figure 4A**).

Three innate-like T cell clusters (C13, C18, and C19) characterized by high expression of FCER1G were identified within the CD8A-dominant group (**Figure 4A**). Reduced expression of CD8B in these clusters (**Figure 4B**) supports their innate-like phenotype as murine and human CD8^+^ innate-like T cells (i.e. MAIT cells, iNKT cells, and γδ T cells) show predominant expression of the CD8αα homodimer, in contrast to conventional T cell populations that are mainly CD8αβ^+^ (47, 48). The transcription profile of C13 and C18 (but not C19) indicated high cytotoxic potential (FASLG^+^, PRF1^+^, GZMA^+^, GZMB^+^, GZMK^+^) (**Figure 4A**). FCER1G provides necessary activation motifs for several NK receptors, including the natural cytotoxicity receptor 3 (NCR3) (49). However, expression of NCR3 was not restricted to FCER1G^+^ clusters of canine TCRαβ^+^ T cells (C13, C18, and C19) but was also detected in C7 (terminal effector CTL), C14 (central memory CTL), and C16 (progenitor exhausted, see below) (**Figure 4A**), probably due to different signal transduction pathways including FCER1G and/or CD3ζ adaptor molecules (49). Similarly, widespread expression of NCR2 encoding NKp44 was observed among CD8A-dominant clusters (**Figure 4A**). In contrast, NKG2C was only up-regulated in the two CD8^+^ FCER1G^+^ innate-like clusters with high cytotoxic potential (C13 and C18) (**Figure 4A**). Besides the similarities as compared to C18, C13 specifically showed upregulated expression of TRBV25-1, TRBV28, and CXCR6 (**Figure 4A**), indicating a mucosa-associated invariant T-cell (MAIT)-like phenotype (50, 51). Of note, V(D)J analysis confirmed a highly restricted T cell receptor repertoire of the MAIT-like cluster C13 (see below). C18 represents a population of canine CD8^+^ FCER1G^+^ ZEB2^+^ innate-like effector cells (**Figure 4A**). The third FCER1G^+^ innate-like cluster of canine TCRαβ^+^ CD8A^+^ T cells C19 was classified as iNKT-like due to elevated expression of TRAV24 but lack of typical cytotoxic effector molecules (**Figure 4A**). Down-regulated transcription of CD5 is another specific characteristic of the canine iNKT-like cell type (**Figure 4A**). Furthermore, enhanced expression of the MHC-II-encoding gene DLA-DQA1 as well as absence of KLRB1 expression was detected in cells of this cluster (**Figure 4A**). In humans, this iNKT-phenotype is associated with exhaustion and decreased expression of cytotoxic factors (52). Accordingly, canine iNKT-like cells showed an elevated transcription level of TOX (**Figure 4A**), known to promote the generation of exhausted T cells while repressing development of the KLRG1^+^ T effector cell lineage (53–55). Cluster C16 differs from the iNKT-like cluster C19 in terms of FCER1G and TRAV24 expression but shows a similar progenitor exhausted T cell profile with upregulated transcription of TOX, TCF7 (encoding the transcription factor TCF-1), and CD244 (encoding the inhibitory receptor 2B4) and downregulated effector genes, including ZEB2, KLRG1, GZMA, GZMB, GZMK, PRF1, and FASLG (53–55). Furthermore, C16 (progenitor exhausted) shows highest expression of CXCR3 among CD8A-dominant clusters and constitutive expression of IFNG (**Figure 3B**; compare **Figure 4A** for cluster location of C16).

Taken together, scRNA-seq revealed new insights into the heterogeneity of canine TCRαβ^+^ T cells and led to the first identification of CD8^+^ FCER1G^+^ innate-like T cell populations in dogs, including a MAIT-like and an innate effector-like cell type, both with high cytotoxic potential as well as an iNKT-like cell type with features of exhaustion.

### 3.4 TCRαβ^+^ CD4/CD8A-mixed clusters of the dog include terminally exhausted and proliferating cells

**Figure 5:**
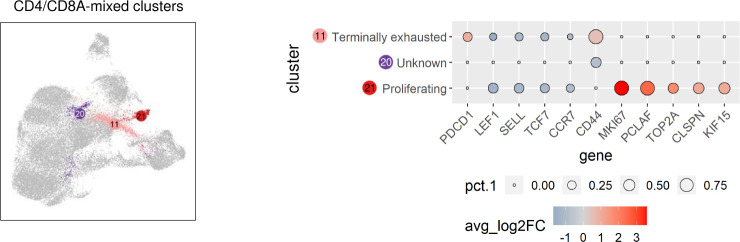
TCRαβ^+^ CD4/CD8A-mixed clusters of the dog include terminally exhausted and proliferating cells. CD4/CD8A-mixed clusters highlighted in UMAP plot were annotated based on expression of signature genes shown in Dot plot. The dot size corresponds to the percentage (pct.1) of cells expressing the gene in each CD4/CD8A-mixed cluster, the color represents the expression level (average_log2 fold change).

Significantly upregulated expression of PDCD1 encoding programmed cell death protein-1 (PD-1) was exclusively found in C11 (**Supplementary Table 2**), representing a CD4/CD8A-mixed cluster (**Figure 2C**). Thus, C11 is likely to comprise canine TCRαβ^+^ terminally exhausted CD4 and CD8 T cells (**Figure 5**). Due to the lack of lineage-defining signature transcripts, C20 was designated “unknown” CD4/CD8A-mixed population. Cells of this cluster express comparatively low levels of naïve markers (LEF1, SELL, TCF7, CCR7) as well as of activation markers (e.g. CD44) (**Figure 5** and **Supplementary Table 2**). Cells in C21 uniquely expressed numerous cell cycle-related genes, such as MKI67, PCLAF, TOP2A, CLSPN, and KIF15, and accordingly C21 contains proliferating TCRαβ^+^ T cells (**Figure 5** and **Supplementary Table 2**).

### 3.5 Canine iNKT-like cells frequently have more than one TRA rearrangement

**Figure 6:**
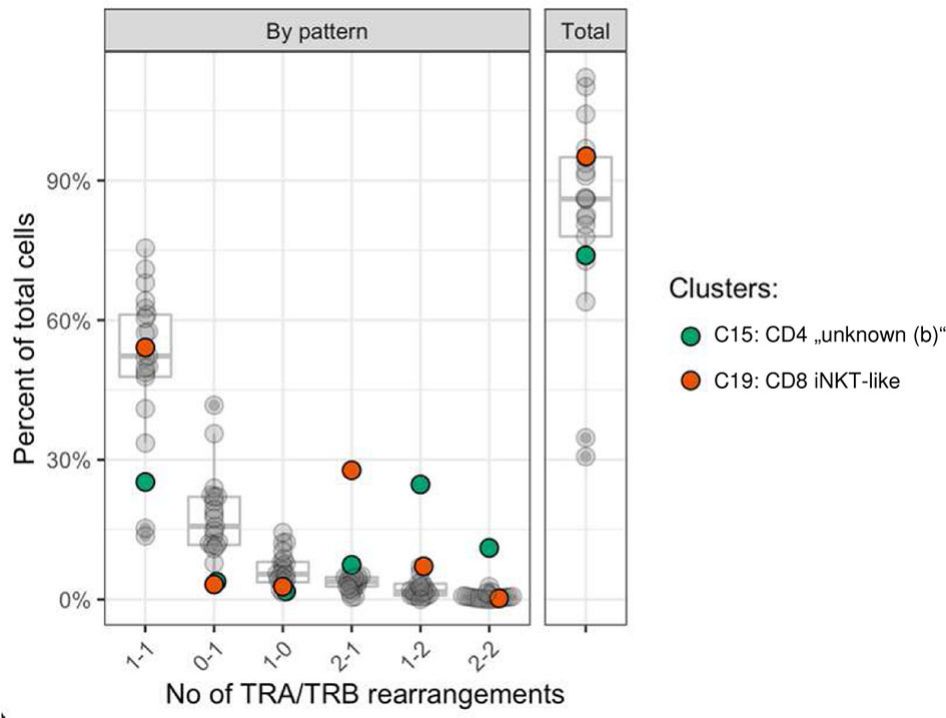
Frequency of TRA/TRB rearrangement patterns per cell stratified by cluster. Most cells had one TRA and one TRB rearrangement (median 52.1%). Cells with 2xTRA/1xTRB rearrangements were more common in the CD8A-dominant iNKT-like cluster (C19, red) and cells with either 1xTRA/2xTRB rearrangements or 2xTRA/2xTRB rearrangements were more common in the CD4-dominant “unknown” (b) cluster (C15, green). x-axis: number of TRA and TRB rearrangements per cell, format: ‘x-y’; x: number of TRA rearrangements; y: number of TRB rearrangements per cell; example: ‘2-1’: cells with 2 TRA rearrangements and 1 TRB rearrangement; y-axis: percent of cells out of all cells.

The T cell receptor repertoire was characterized for all clusters identified by transcriptome analysis. As expected, the majority of cells had one productive TRA and TRB rearrangement each (16,603 cells, 62.1%) followed by cells with either one productive TRB rearrangement only (5,640 cells, 21.1%) or one productive TRA rearrangement only (2,337, 8.7%) (**Supplementary Table 6**). The medium percentage of cells for which at least one rearrangement could be detected was 87.4% with 17/22 clusters showing TCR rearrangements in at least 75% of cells (**Figure 6**). Some clusters comprised a disproportionate number of cells with more than two rearrangements. In the CD8A-dominant iNKT-like cluster, 26.6% of cells had 2xTRA/1xTRB rearrangements (median percentage across all clusters 3.8%). Furthermore, in the CD4-dominant cluster C15, designated “unknown (b)” (**Figure 3A**), 21.7% of cells had 1xTRA/2xTRB rearrangements (median percentage across all clusters 1.7%) and 9.7% of cells had 2xTRA/2xTRB rearrangements (median percentage across all clusters 0.5%) (**Figure 6**). For the dog, this is the first indication of dual TCR expression, previously observed in murine and human studies (56).

### 3.6 MAIT-like and iNKT cells have a skewed TRA junctional length

**Figure 7:**
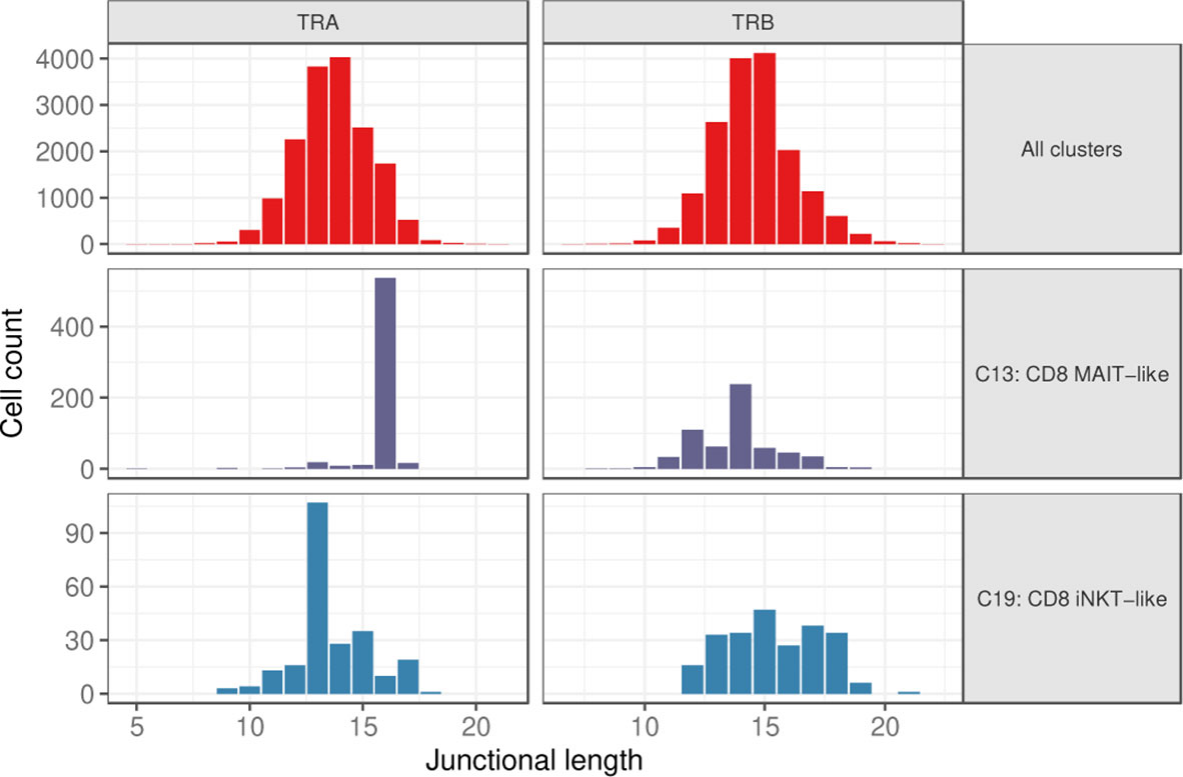
Junctional length of TRA and TRB rearrangements for all clusters combined (top), the MAIT-like cluster (C13) (middle), and the iNKT-like cluster (C19) (bottom).

Considering cells with a single TRA and TRB rearrangement each, the junctional length of TRA and TRB rearrangements was normally distributed for most clusters with a median length of 14 and 15 amino acids (aa), respectively (**Figure 7** and **Supplementary Figure 3**). Clusters with a skewed junctional length were the MAIT-like cluster (C13), which had a markedly restricted TRA junctional length at 16 aa, as well as the iNKT-like cluster (C19) with a dominant TRA junctional length of 13 amino acids (**Figure 7** and **Supplementary Figure 3**). MAIT-like cells had a slight bias toward 12 aa and 14 aa in the TRB repertoire, but overall, the TRB junctional length was less skewed than the TRA junctional length. Since TRA rearrangements are not routinely assessed in dogs, these data provide the first evidence for invariant T cell receptors in dogs.

### 3.7 TRAV9-11 is preferentially used in FCER1G^+^ innate-like CD8A T cell subsets and rearranges almost exclusively to TRAJ28 in MAIT-like cells

**Figure 8:**
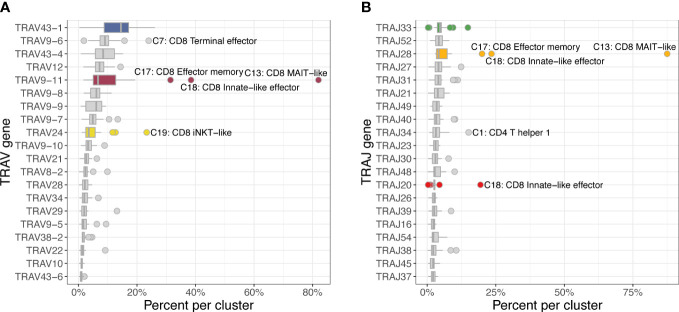
Usage frequencies of the 20 most common TRAV genes **(A)** and TRAJ genes **(B)** showing usage bias for several subsets of the CD8A-dominant group (CD8). TRAV9-11 was more frequently used in MAIT-like cells (C13) and, to a lesser degree, in innate-like effector (C18) and effector memory (C17) cells. TRAJ28 was disproportionally utilized in MAIT-like cells (C13).

The most frequently used TRAV gene families were TRAV43 and TRAV9 with the top 3 TRAV genes having a median percent usage of 14.4% (TRAV43-1), 8.9% (TRAV9-6), and 8.3% (TRAV43-4) across 22 clusters (**Figure 8**). A clear outlier was TRAV9-11, which was used in 82.5% of MAIT-like cells (C13), 35.4% of innate-like effector cells (C18) and 28.2% of effector memory cells (C17) of the CD8A-dominant group. The TRAJ usage was generally evenly distributed with the three most frequently used genes TRAJ33, TRAJ52 and TRAJ28 accounting for 4-5% of rearrangements. However, analogous to TRAV genes, MAIT-like cells (C13) showed a strong bias utilizing TRAJ28 in 87.7% of cells.

**Figure 9:**
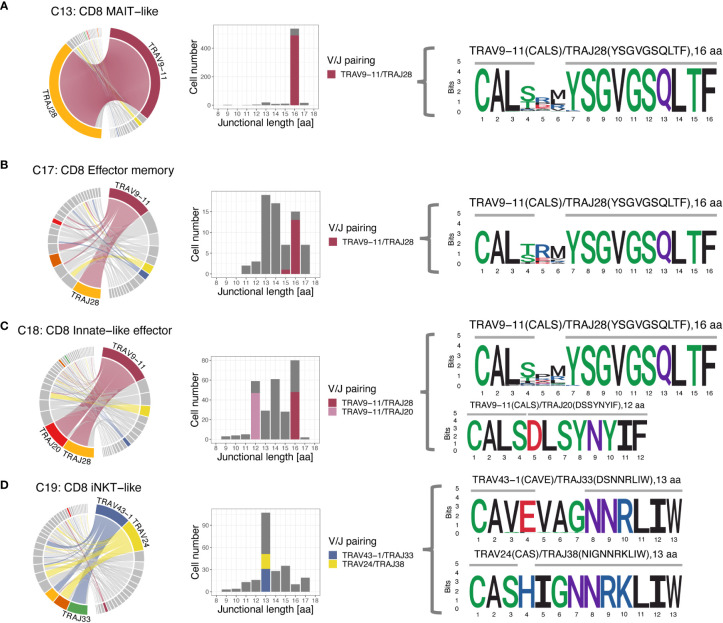
T cell receptor alpha (TRA) V/J pairing (left), junctional length (middle) and junctional sequence (right) of 4 clusters with skewed V/J usage. **(A-C)** The MAIT-like cluster (C13), and to a lesser degree, the effector memory (C17) and innate-like effector (C18) clusters all have a dominant TRAV9-11/TRAJ28 rearrangement with 16 aa junctional length and the CDR3 is mostly germline encoded with two amino acids added. **(C)** The TRAV9-11 gene in the innate-like cluster (C18) additionally rearranges to TRAJ20 with 12 aa junctional length, which is entirely germline encoded and has no added bases. **(D)** The iNKT-like cluster (C19) predominantly uses the V genes TRAV43-1 and TRAV24, which rearrange to a number of different J genes. While 13 aa length sequences dominate, the junctional sequences are more diverse than sequences utilizing TRAV9-11 in the other 3 clusters and show more evidence of deletions and additions. Left column: chord diagram displaying V/J pairing; Middle column: junctional length; dominant V/J pairings are highlighted; Right column: position weight matrix of junctional sequence; horizontal grey bar indicates the extent of the germline variable (right) and joining (left) genes.

When assessing V/J pairing, 82.1% of MAIT-like cells (C13) and 21.2% of effector memory cells (C17) of the CD8A-dominant group utilized TRAV9-11 rearranged to TRAJ28 (**Figure 9**). In CD8A innate-like effector cells (C18), the dominant V gene TRAV9-11 also rearranged to TRAJ28 (15.1% of cells) but additionally paired with TRAJ20 at slightly higher frequency (16.8% of cells). In iNKT-like cells (C19), the two dominant V genes TRAV43-1 and TRAV24 rearranged to a variety of different J genes, suggesting a less restricted pairing pattern in this subset.

### 3.8 FCER1G^+^ innate-like CD8A T cells have variable diversification of TRA junctional regions

Given the disproportionate use of TRAV9-11 in MAIT-like (C13), innate-like effector (C18), and effector memory (C17) cells of the CD8A-dominant group we further explored the junctional sequence in these clusters. Most cells had a junctional length of 16 amino acids (568 cells, 92.1%) and, except for amino acid positions 4-6, an invariant junctional sequence with the motif ‘CAL… YSGVGSQLTF’ (**Figure 9**). This was a consistent finding in all 3 examined clusters and, at least for MAIT-like cells (C13), was true for all 4 dogs. Out of the 16 aa positions, 10 were encoded by the J gene, 4 by the V gene, and 2 were added nucleotides. A subset of CD8A-dominant innate-like effector cells (C18) had a junctional length of 12 amino acids (49 cells, 7.7%). The subset of CD8A-dominant innate effector cells (C18) with a junctional length of 12 amino acids (7.7%) likely reflects a single expanded clone, as it was found only in dog 4, was supported by only 49 cells, and had a single junctional sequence (motif ‘CALSDLSYNYIF’).

### 3.9 TRB repertoire bias is most common in MAIT-like and iNKT-like cells but is less pronounced than in TRA

**Figure 10:**
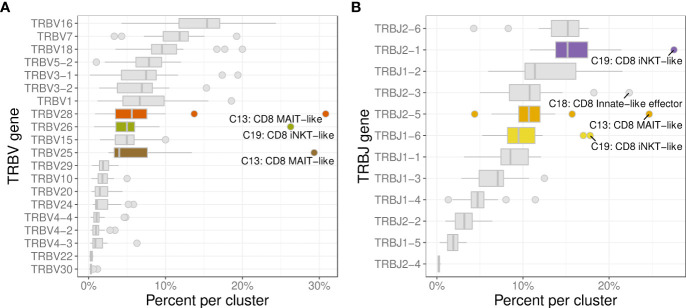
Usage frequencies of the most common TRBV genes **(A)** and TRBJ genes **(B)** showing usage bias for MAIT-like cells (C13), and iNKT-like cells (C19).

**Figure 11:**
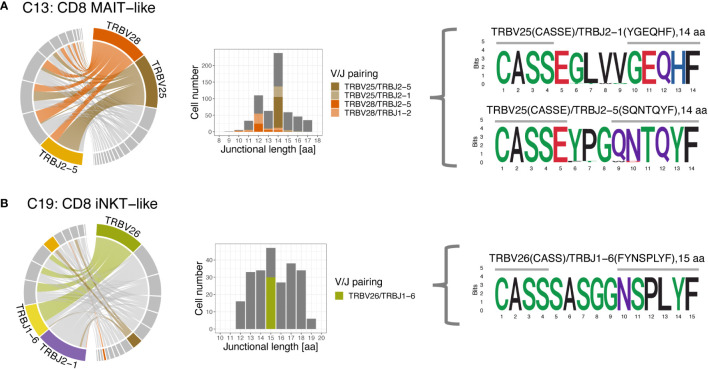
T cell receptor beta (TRB) V/J pairing (left), junctional length (middle) and junctional sequence (right) of two clusters with skewed V/J usage. **(A)** The MAIT-like cluster predominantly uses TRBV25 and TRBV28, which rearrange to a variety of J genes. Rearrangements involving TRBV25 and TRBV28 mostly have a junctional length of 14 and 12 aa, respectively. **(B)** The iNKT-like cluster preferentially uses TRBV26 and rearranges to various J genes. Rearrangements utilizing TRBV26 have variable junctional length; note low coverage for this cluster. Left column: chord diagram displaying V/J pairing; Middle column: junctional length; dominant V/J pairings are highlighted; Right column: position weight matrix of junctional sequence; horizontal grey bar indicates the extent of the germline variable (right) and joining (left) genes.

The 3 most frequently utilized TRBV genes were TRBV16 (14.9%), TRBV7 (11.9%), and TRBV18 (10.2%) (**Figure 10A**). Clusters with a strongly biased gene usage were the MAIT-like cluster (C13) utilizing TRBV28 in 30.7% and TRBV25 in 30.2% of cells and the iNKT-like cluster (C19) utilizing TRBV26 in 27.9% of cells. Owing to a smaller number of J genes, the median TRBJ gene usage was generally higher than that of TRAJ genes with TRBJ2-6, TRBJ2-1, and TRBJ1-2 used in 15.2%, 14.8%, and 11.1% of clusters, respectively (**Figure 10B**). Cells utilizing a TRBJ2 gene were slightly more abundant than cells using a TRBJ1 gene (44.1% vs. 55.9%). Disproportionately used TRBJ genes were TRBJ2-1 in iNKT-like cells (26.1%) and TRBJ2-5 in MAIT-like cells (25.0%). The V/J gene pairing for TRB sequences was a lot less restricted than observed for TRA (**Figure 11**). Any given TRB V gene rearranged to multiple different J genes at similar frequencies.

### 3.10 A MAIT-like dominated TRA supercluster is characterized by large size, high centrality and high publicity

**Figure 12:**
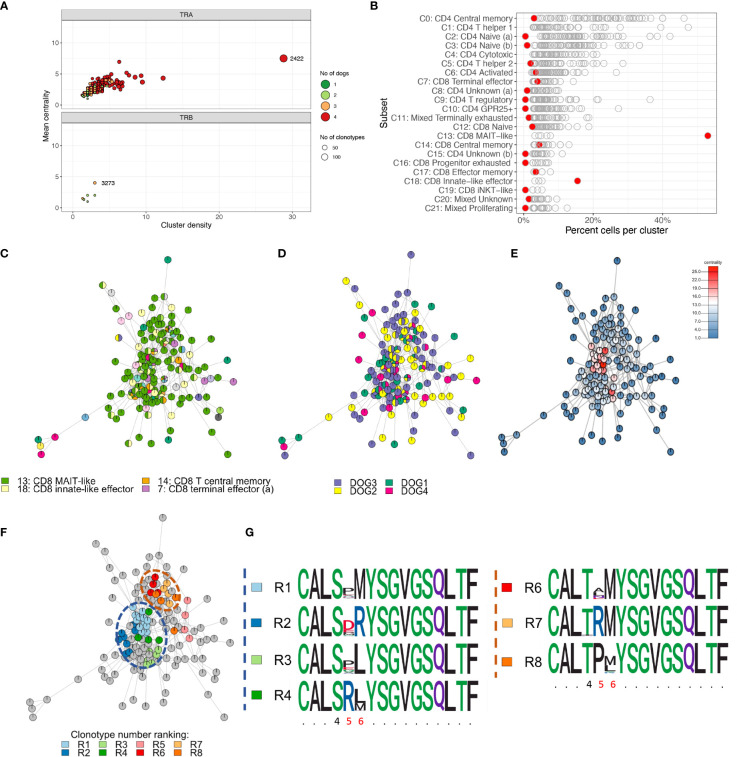
Cluster analysis identifying closely related clonotypes across T cell subsets and dogs. **(A)** Cluster summary. TRA cluster #2422 stands out because it comprises the most unique clonotypes, the highest cluster density and centrality and comprises sequences from all four dogs (top). There are markedly fewer TRB clusters than TRA clusters. TRB clusters contain fewer clonotypes, have a lower cluster density and centrality and comprise sequences from at most three dogs. **(B)** Cluster composition by T cell subset. Each point is one cluster, cluster #2422 is highlighted red. MAIT-like cells (C13) are overrepresented in cluster #2422; iNKT-like cells (C19) are not enriched. **(C-F)** Network plots of ‘relatedness’ of clonotypes in TRA cluster #2422 highlighting different variables of interest. All clonotypes that differ by one amino acid are connected by edges. Pie chart notes depict the relative frequency of values for a given variable. **(C)** T cell subsets. MAIT-like cells (green) are dominant; certain clonotypes are seen in multiple different subsets. **(D)** Dogs. The degree of ‘publicity’ of clonotypes ranges from unique for a given clonotype to shared by all four dogs; **(E)** Centrality. The centrality rank reflects the number of edges for a given node. The high ranking clonotypes in the central areas of the cluster are indicative of a high ‘density’ of closely related sequences; **(F)** Sub-clusters derived by complete linkage (cl) clustering highlighting the most closely related sequences. All clonotypes within a given cl cluster differ by a single amino acid only; the top-ranking cl clusters based on clonotype abundance (rank R1-R8) are colored. **(G)** Dissecting the sequence composition of cl clusters highlighted in **(F)** using position weight matrixes. Two groups of cl clusters (blue and orange dashed circle) are highly variable in positions 5 and 6 and have either a serine (S) (blue group) or threonine (T) (orange circle) in position 4. x-axis: amino acid position, invariable positions are abbreviated with a period, variable positions within a cl cluster are highlighted red.

To characterize repertoire overlap across T cell subsets and dogs, we clustered clonotypes with similar amino acid sequence. The TRA repertoire had a lower number of unique junctional sequences (clonotypes) (n=9,542) but a markedly higher number of clusters (n=985) as compared to the TRB repertoire (11,150 clonotypes; 168 clusters) (**Figure 12A**). For TRA, cluster # 2422 (TRAV9/TRAJ28) stood out because it had the most cells (n=625), the most clonotypes (n=144), the highest centrality and the greatest cluster density (**Figure 12A**). Furthermore, this cluster was predominantly comprised of MAIT-like cells (C13) and, to a lesser degree, innate-like effector cells (C18) of the CD8A-dominant group (**Figure 12B**). In contrast, other dominant clusters consisted of multiple T cell subsets that were mostly of CD4 lineage (data not shown). For TRB, cluster #3273 had the highest centrality and cluster density (**Figure 12A**), however, all metrics were significantly lower than those of TRA cluster # 2422. While 16.5% of TRA clusters were comprised of clonotypes from all 4 dogs, no TRB cluster had sequences from more than 3 dogs (**Figure 12A**). TRB clusters with clonotypes from 3 dogs only comprised 4.2% of all clusters (**Figure 12A**). When assessing the composition of clusters with respect to T cell subset, cluster #2422 stood out because it was primarily composed of CD8 MAIT-like cells (**Figures 12B, C**). Visualizing clonal relationships using network plots, we found that certain clonotypes occurred in more than one T-cell subset (**Figure 12C**), were unique to one dog or were shared by multiple dogs (**Figure 12D**), and were extremely related with up to 25 sequences that differed by a single amino acid only (**Figure 12E**). The sequence variability was restricted to amino acid positions 4-6 (**Figures 12F, G**). When comparing repertoire overlap across T cell subsets, we found the greatest overlap between subsets of the CD4-dominant group, specifically T central memory (C0), naïve (a) (C2) and naïve (b) (C3) cells (**Supplementary Figure 4**). The CD8A-dominant MAIT-like subset shared the least overlap with other T cell subsets (**Supplementary Figure 4**), despite harboring the largest cluster (**Figure 12**).

## 4 Discussion

Given the value of the dog as a model species for various human disorders (including autoimmune diseases, allergy, and cancer), a better understanding of canine T cells could support in-depth mechanistic analyses in these models and might reveal novel targets for immunotherapy. Here, we use 5’ scRNA-seq combined with immune repertoire sequencing to resolve TCRαβ^+^ T cell populations of healthy dogs at an unprecedented resolution. The resulting T cell atlas of canine peripheral blood sheds new light into the diversity of these critical immune cells. Among the 22 annotated clusters were several CD4 and CD8A T cell populations not previously described in dogs. Of note, high expression of FCER1G identified three populations of canine innate-like CD8A^+^ T cells in peripheral blood, showing a MAIT-like, an innate effector-like, and an iNKT-like phenotype, respectively. Expression of FCER1G has been shown to be associated with human innate T cells, comprising MAIT cells, iNKT cells and γδ T cells (57). These cells exhibit innate characteristics during inflammation and infection, such as rapid activation kinetics without prior pathogen exposure, and the capacity for TCR-independent activation by inflammatory cytokines such as IL-12, IL-18, and type I interferons (57–60). Expression of gene products responsible for effector functions such as cytotoxicity and cytokine production is a hallmark of human innate T cells (57). In the current study, high cytotoxic potential was observed in the MAIT-like as well as in the CD8A^+^ innate effector-like cell populations. Of note, innate effector-like T cells may be interesting candidates for novel cancer treatment strategies. Recently, FCER1G^+^ NK1.1^+^ innate-like TCRαβ^+^ T cells with high cytotoxic potential have been identified in murine and human malignancies. Activation of IL-15 signaling in corresponding murine progenitor cells was shown to suppress tumor-growth *in vivo* after adoptive transfer (61). Furthermore, a population of IL-15-induced human circulating NKp30^+^FcϵR1γ^+^ CD8^+^ T cells was described to exhibit high NK-like anti-tumor activity *in vitro* and in a preclinical xenograft mouse model *in vivo* (62). FCER1G-dependent upregulation of NK receptors may potentiate rapid acquisition of effector functions of innate-like T cells in tumor tissues (61). Interestingly, in the present study we found widespread expression of several NK receptors among canine CD8A^+^ T cells, including the FCER1G^+^ populations. Our data provide an important reference for future studies evaluating T cell responses in canine diseases. This also applies, for example, to the newly identified canine TOX^+^TCF7^+^ progenitor-like exhausted T cell population (C16). Exhausted CD8^+^ T cells are the major target of checkpoint blockade in patients with cancer and modulation of TOX, a transcription factor required for epigenetic remodeling and survival of functionally impaired exhausted T cells, has been suggested as a potential target for immunotherapy (63). It needs to be studied, whether TOX^+^ TCF7^+^ progenitor-like exhausted T cells are enriched in tumors of dogs.

The integration of immune repertoire sequencing into the single cell RNA seq workflow enabled the discovery of MAIT-like T cells in dogs. Similar to MAIT cells of other species (64), the newly identified MAIT-like cell population in dogs expresses an invariant TCR-α chain characterized by a distinct TRAV/TRAJ gene usage and a constant junctional length, paired with a TCR-β chain using a limited number of TRBV genes. Interestingly, dogs lack the nonclassical MHC-I-like molecule MR1, which presents microbial riboflavin precursor derivatives to MAIT cells in most mammals, as well as the TRAV1 gene, the canonical TRAV gene used in these cells (64). Instead, canine MAIT-like cells show preferential usage of the TRAV9-11 gene. Thus, the kind of antigens recognized by MAIT-like cells of dogs as well as the mechanism of antigen presentation are exciting questions for future studies.

Similar to MAIT-like cells, canine FCER1G^+^ innate-like iNKT-like cells, showed a biased TRA repertoire with a restricted junctional length but two different dominant V genes, TRAV24 and TRAV43-1. In a previous study, a canine CD3^+^ T cell population reactive to α-galactosylceramide (α-Gal-Cer)-loaded mouse CD1d has been suggested to represent iNKT cells of the dog with homology of the variable and joining regions of the TCRα-chain to both, mouse Vα14-Jα281 and human Vα24-JαQ (65). However, CD1d, the non-classical MHC class-I-related molecule presenting (glyco)lipid antigens to iNKT cells in various species, is likely non-functional in dogs (66, 67). Thus, whether dogs utilize an alternative MHC-I-like molecule than CD1d presenting a distinct set of antigens to the newly identified innate-like iNKT-like population, needs further investigation. In humans, the MHC-II^+^KLRB1^-^ phenotype of iNKT cells is not only associated with decreased expression of cytotoxic molecules, which was also observed in this study for dogs but additionally with a Th1-skewed cytokine profile (52). In future studies, scRNA-seq analysis following stimulation could provide further insights into cytokine-producing capabilities and functional properties of the various TCRαβ^+^ T cell subpopulations of dogs. This is expected to also reveal a cluster of CD4^+^ Th17 cells. In the present study, the master transcription factor of Th17 cells (encoded by RORC), as well as other Th17 cell-related molecules (STAT3, CCR6, RORA, IL23R, IL17A) were not found to be differentially expressed between clusters (data not shown). The combined analysis of transcriptome and immune repertoire of TCRαβ^+^ T cells established in this study can be also used in further experiments to characterize tissue-specific expression of effector and regulatory molecules as well as potentially distinct TCR repertoires of circulating vs. tissue-resident T cells, including Treg cells.

Canine TCRαβ^+^ T cells comprise a fraction of ~15% non-conventional CD4^-^CD8α^-^ double-negative (14) as well as a small fraction of non-conventional CD4^+^CD8α^+^ double-positive T cells (68–70). Double-negative T cells were excluded from analysis in the present study since it was not possible to discriminate between true double-negative T cells and double-negative T cells resulting from drop-out of CD4, a phenomenon already observed previously (5, 13). This could also have resulted in an underestimation of CD4^+^CD8A^+^ dp T cells. Single cell RNA-seq of sorted dn/dp TCRαβ^+^ T cells in future experiments would allow for comparison of gene expression patterns of these non-conventional populations with conventional single-positive T cells. Of note, presence of well-known T helper cell and Treg cell clusters in the CD4-dominant group supports the classification used in the present study.

Taken together, this study contributes to a better understanding of the cellular diversity of peripheral blood TCRαβ^+^ T cells providing a basis to inform translational efforts in the field of immunotherapy.

## Data availability statement

The data presented in the study are deposited in the NCBI GEO repository, accession number GSE218355.

## Ethics statement

The animal study was reviewed and approved by Saxony State Office (Landesdirektion Sachsen) in Leipzig, Germany (approval number: DD24.1-5131/444/30).

## Author contributions

ME, GA, and SK designed experiments and wrote the manuscript. ME and SK performed experiments. PM provided key reagents. HC performed the TCR (TRA, TRB) cluster analysis. All authors contributed to the article and approved the submitted version.
