# Supplementary figures and images for: Canine peripheral blood TCRαβ T cell atlas: Identification of diverse subsets including CD8A+ MAIT-like cells by combined single-cell transcriptome and V(D)J repertoire analysis
Source: Front Immunol. 2023 Feb 23;14:1123366. doi: 10.3389/fimmu.2023.1123366 (PMC9995359; doi:10.3389/fimmu.2023.1123366)

## Slide 1
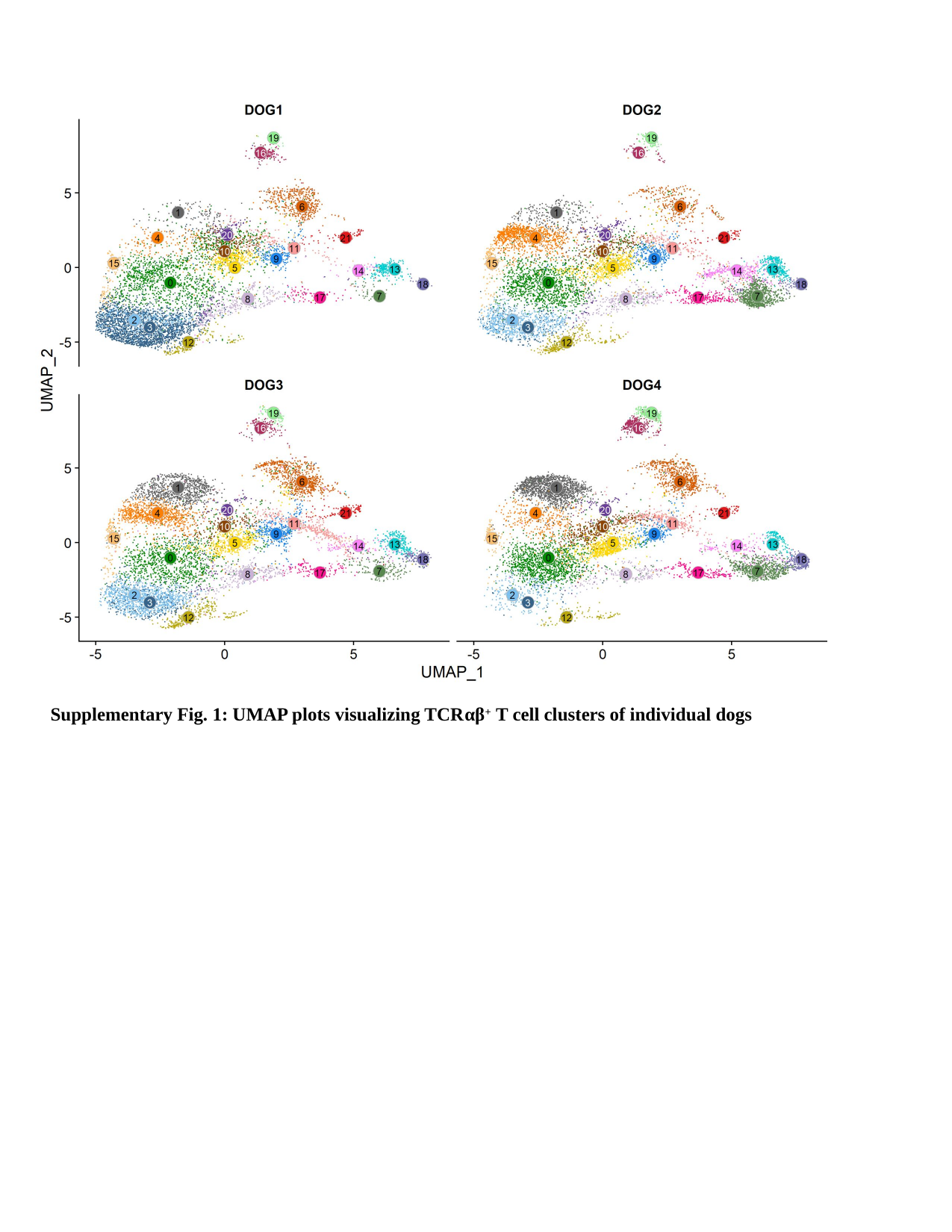

Supplementary Fig. 1: UMAP plots visualizing TCRαβ+ T cell clusters of individual dogs

Supplement: Supplementary file 1 [file Presentation_1.pptx]
