# Supplementary material for: Canine peripheral blood TCRαβ T cell atlas: Identification of diverse subsets including CD8A+ MAIT-like cells by combined single-cell transcriptome and V(D)J repertoire analysis
Source: Front Immunol. 2023 Feb 23;14:1123366. doi: 10.3389/fimmu.2023.1123366 (PMC9995359; doi:10.3389/fimmu.2023.1123366)
Supplement: Supplementary file 2 [file Presentation_2.pptx]

## Slide 1
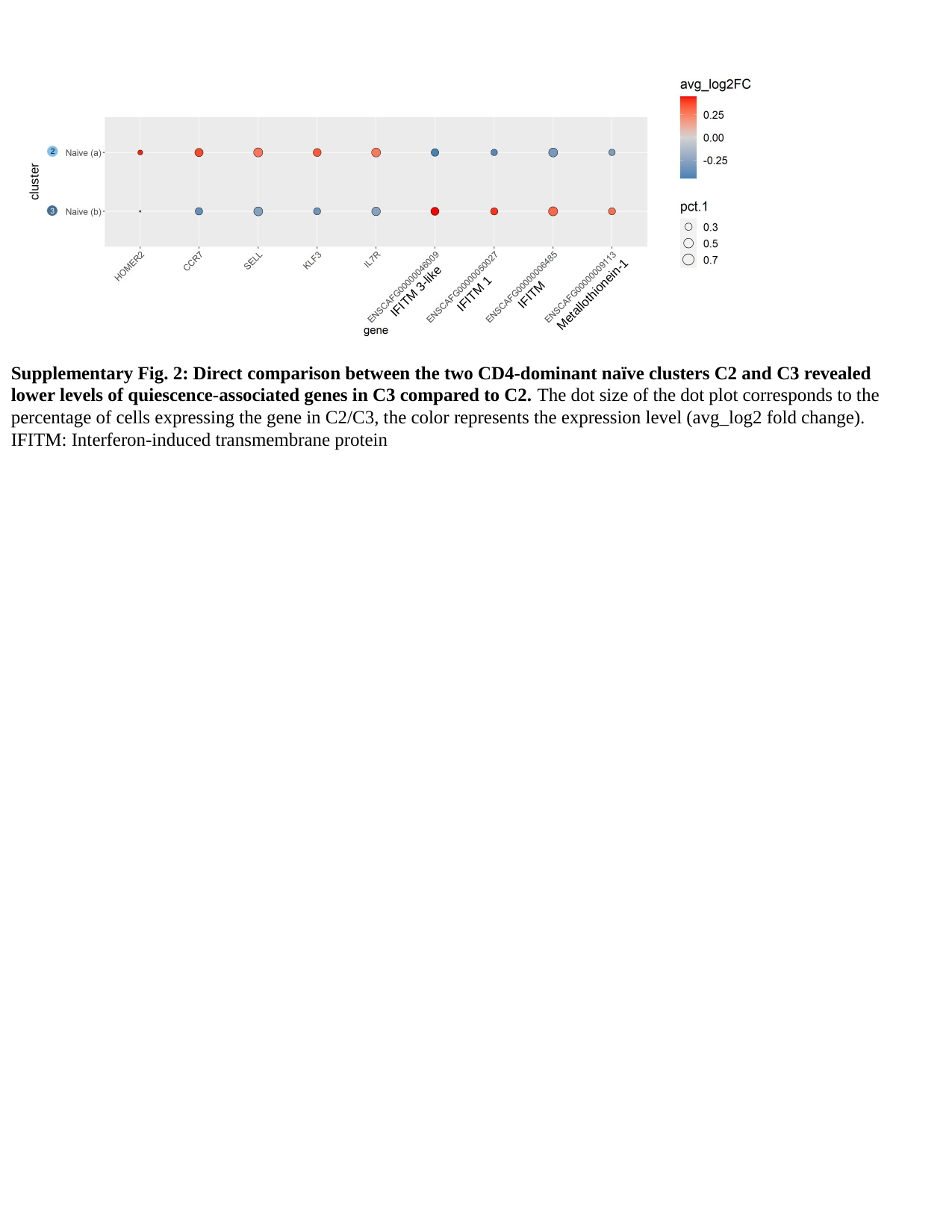

2
cluster
3
Metallothionein-1
IFITM 3-like
IFITM 1
IFITM
Supplementary Fig. 2: Direct comparison between the two CD4-dominant naïve clusters C2 and C3 revealed lower levels of quiescence-associated genes in C3 compared to C2. The dot size of the dot plot corresponds to the percentage of cells expressing the gene in C2/C3, the color represents the expression level (avg_log2 fold change). IFITM: Interferon-induced transmembrane protein
