# Supplementary material for: Canine peripheral blood TCRαβ T cell atlas: Identification of diverse subsets including CD8A+ MAIT-like cells by combined single-cell transcriptome and V(D)J repertoire analysis
Source: Front Immunol. 2023 Feb 23;14:1123366. doi: 10.3389/fimmu.2023.1123366 (PMC9995359; doi:10.3389/fimmu.2023.1123366)
Supplement: Supplementary file 3 [file Presentation_3.pptx]

## Slide 1
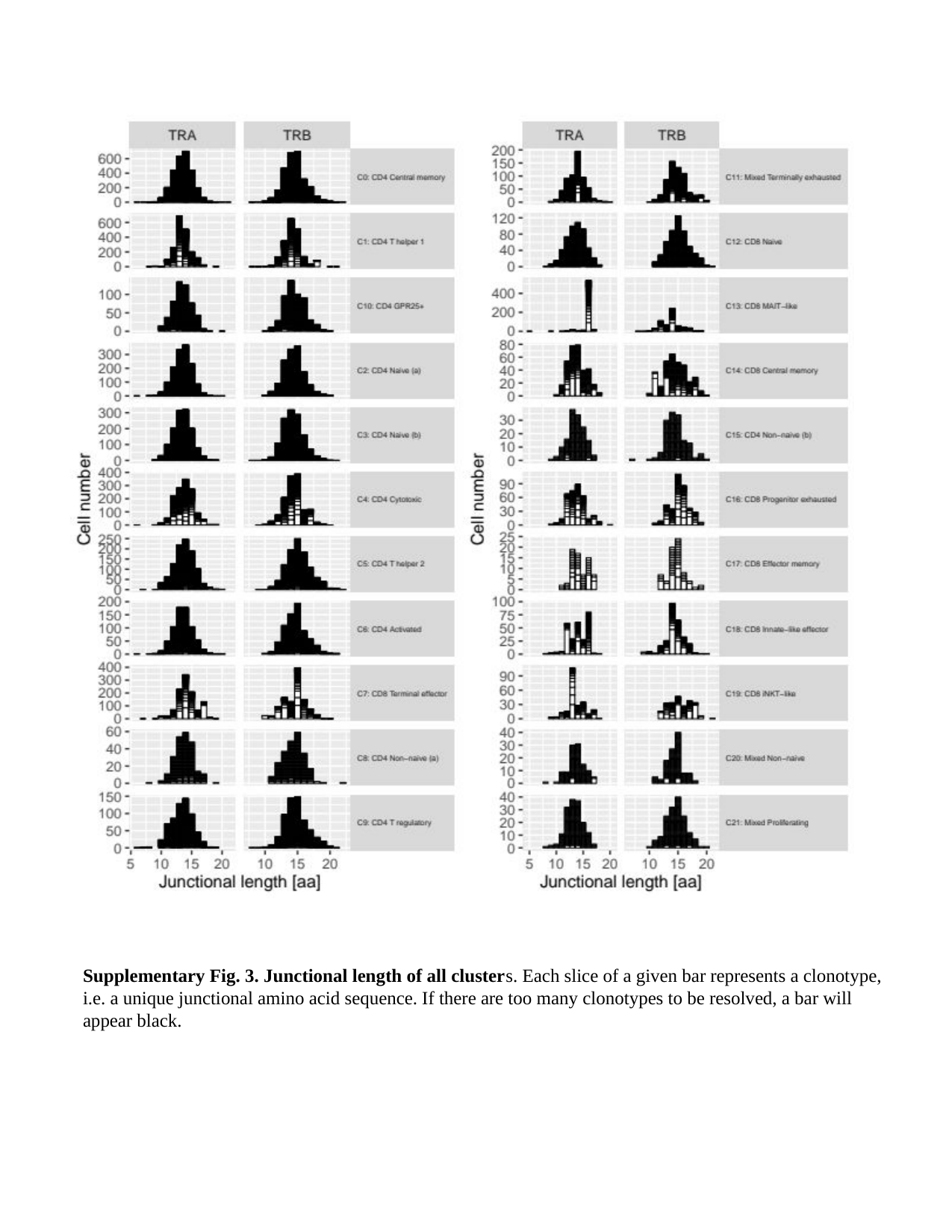

Supplementary Fig. 3. Junctional length of all clusters. Each slice of a given bar represents a clonotype, i.e. a unique junctional amino acid sequence. If there are too many clonotypes to be resolved, a bar will appear black.
