# Supplementary material for: Canine peripheral blood TCRαβ T cell atlas: Identification of diverse subsets including CD8A+ MAIT-like cells by combined single-cell transcriptome and V(D)J repertoire analysis
Source: Front Immunol. 2023 Feb 23;14:1123366. doi: 10.3389/fimmu.2023.1123366 (PMC9995359; doi:10.3389/fimmu.2023.1123366)
Supplement: Supplementary file 4 [file Presentation_4.pptx]

## Slide 1
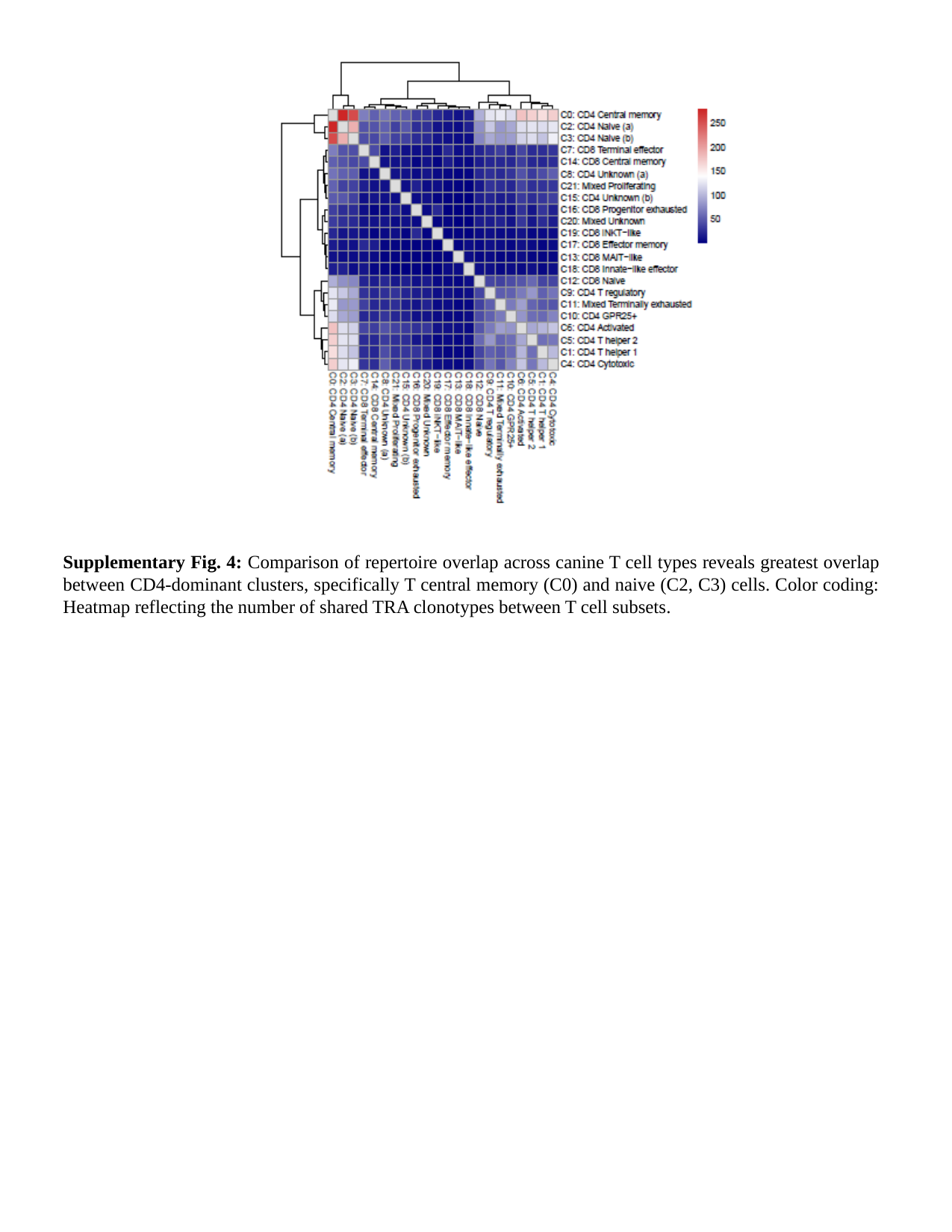

Supplementary Fig. 4: Comparison of repertoire overlap across canine T cell types reveals greatest overlap between CD4-dominant clusters, specifically T central memory (C0) and naive (C2, C3) cells. Color coding: Heatmap reflecting the number of shared TRA clonotypes between T cell subsets.
