# Supplementary material for: Canine peripheral blood TCRαβ T cell atlas: Identification of diverse subsets including CD8A+ MAIT-like cells by combined single-cell transcriptome and V(D)J repertoire analysis
Source: Front Immunol. 2023 Feb 23;14:1123366. doi: 10.3389/fimmu.2023.1123366 (PMC9995359; doi:10.3389/fimmu.2023.1123366)

Supplementary Material

**Supplementary Table 1: Primer sequences adapted for amplification of canine T cell receptor alpha (TRA) and T cell receptor beta (TRB) genes.** Primers were designed using the default settings of the Geneious software primer search function (Version 2022.1.1). The reverse primers were designed to reflect the approximate position and biochemical features (Tm) of the primers described in the human and mice Chromium Single Cell V(D)J Reagent kit (version CG000086). To eliminate amplification bias for the TRB locus based on differential usage of the 2 constant region genes, the TRB reverse primer was chosen to target a nucleotide sequence that is shared by both TRB constant region genes. Adequate PCR amplification was verified by visualizing a smear/curve of the expected size on Bioanalyzer traces.


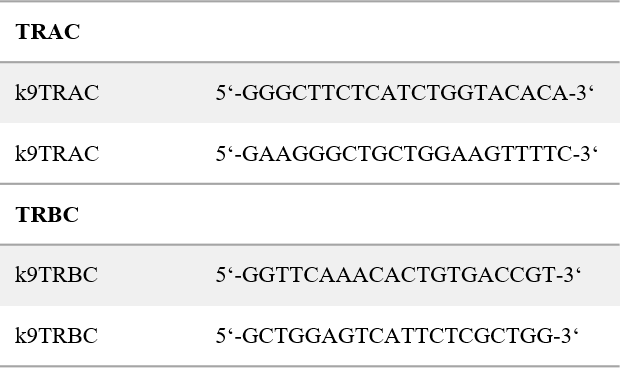

Supplement: Supplementary file 5 [file Table_1.docx]
