# Supplementary material for: Canine peripheral blood TCRαβ T cell atlas: Identification of diverse subsets including CD8A+ MAIT-like cells by combined single-cell transcriptome and V(D)J repertoire analysis
Source: Front Immunol. 2023 Feb 23;14:1123366. doi: 10.3389/fimmu.2023.1123366 (PMC9995359; doi:10.3389/fimmu.2023.1123366)
Supplement: Supplementary file 10 [file Table_6.docx]

Supplementary Material

**Supplementary Table 6: Number and percentage of rearrangements per cell stratified by locus**
